# Mutating alfalfa *COUMARATE 3-HYDROXYLASE* using multiplex CRISPR/Cas9 leads to reduced lignin deposition and improved forage quality

**DOI:** 10.3389/fpls.2024.1363182

**Published:** 2024-03-05

**Authors:** Tezera W. Wolabu, Kashif Mahmood, Fang Chen, Ivone Torres-Jerez, Michael Udvardi, Million Tadege, Lili Cong, Zengyu Wang, Jiangqi Wen

**Affiliations:** ^1^ Institute for Agricultural Bioscience, Oklahoma State University, Ardmore, OK, United States; ^2^ Center for Biotechnology and Genomics, Texas Tech University, Lubbock, TX, United States; ^3^ Queensland Alliance for Agriculture and Food Innovation, The University of Queensland, St. Lucia, QLD, Australia; ^4^ College of Grassland Science, Qingdao Agricultural University, Qingdao, Shandong, China

**Keywords:** CRISPR/Cas9, gene editing, lignin composition, alfalfa, forage quality, MsC3H, nutritional value, digestibility

## Abstract

Alfalfa (*Medicago sativa* L.) forage quality is adversely affected by lignin deposition in cell walls at advanced maturity stages. Reducing lignin content through RNA interference or antisense approaches has been shown to improve alfalfa forage quality and digestibility. We employed a multiplex CRISPR/Cas9-mediated gene-editing system to reduce lignin content and alter lignin composition in alfalfa by targeting the *COUMARATE 3-HYDROXYLASE (MsC3H)* gene, which encodes a key enzyme in lignin biosynthesis. Four guide RNAs (gRNAs) targeting the first exon of *MsC3H* were designed and clustered into a tRNA-gRNA polycistronic system and introduced into tetraploid alfalfa via *Agrobacterium*-mediated transformation. Out of 130 transgenic lines, at least 73 lines were confirmed to contain gene-editing events in one or more alleles of *MsC3H*. Fifty-five lines were selected for lignin content/composition analysis. Amongst these lines, three independent tetra-allelic homozygous lines (*Msc3h-013, Msc3h-121*, and *Msc3h-158*) with different mutation events in *MsC3H* were characterized in detail. Homozygous mutation of *MsC3H* in these three lines significantly reduced the lignin content and altered lignin composition in stems. Moreover, these lines had significantly lower levels of acid detergent fiber and neutral detergent fiber as well as higher levels of total digestible nutrients, relative feed values, and *in vitro* true dry matter digestibility. Taken together, these results showed that CRISPR/Cas9-mediated editing of *MsC3H* successfully reduced shoot lignin content, improved digestibility, and nutritional values without sacrificing plant growth and biomass yield. These lines could be used in alfalfa breeding programs to generate elite transgene-free alfalfa cultivars with reduced lignin and improved forage quality.

## Introduction

Alfalfa (*Medicago sativa*. L) is a perennial legume forage crop widely cultivated in the world for livestock feed as it provides highly nutritious forage (Singer et al., 2018; Chen et al., 2020). Alfalfa is grown for hay, silage, dry pellets, or used directly for grazing as a quality source of proteins for livestock (Zhou et al., 2011; Barros et al., 2019b). The fast-growing perennial growth habit of alfalfa makes it ideal for multiple harvests within one growing season (Singer et al., 2018; Wolabu et al., 2023). The herbaceous nature of alfalfa plants with abundant lignocellulosic biomass yields and the ability to thrive in a variety of soil conditions make it a promising next-generation source of feedstock for biofuels (Hisano et al., 2009; Tong et al., 2015). Alfalfa leaves are more nutritious than stems, since high lignification in stems is a limiting factor to cell wall digestion, and any improvement that helps to liberate nutrients embedded in stems during digestion results in overall benefits for forage animals (Lacefield, 2004; Shadle et al., 2007; Getachew et al., 2018; Wolabu et al., 2023). Therefore, lignin is one of the most important limiting factors for forage nutritional quality (Getachew et al., 2018; Singer et al., 2018; Wolabu et al., 2023). Forage digestibility is negatively correlated with lignin content, which limits the nutritional value of forage crops (Li et al., 2008). Normally, lignin binds with structural carbohydrates (cellulose and hemicellulose) and cell wall proteins, thus reducing nutrient availability (Capstaff and Miller, 2018). Hence, lignin severely affects the digestibility of proteins and other nutritive components (Capstaff and Miller, 2018). Previous studies showed a strong negative correlation between lignin content and sugars released by enzymatic hydrolysis, implying that low lignin content correlates with high released carbohydrate levels (Chen and Dixon, 2007; Ziebell et al., 2010; Tong et al., 2015; Park et al., 2017).

Lignin is one of the most abundant biopolymers and a key structural component crucial for integrity of cell wall and stem strength in plant growth and development (Hisano et al., 2009; Liu et al., 2018; Dixon and Barros, 2019; Vanholme et al., 2019). In plants, lignin plays an important role in protecting plants from adverse environmental stresses, including mechanical damage, drought, low temperature, and resistance to various pests, serving as a mechanical barrier (Li et al., 2008; Bhuiyan et al., 2009; Gallego-Giraldo et al., 2014; Liu et al., 2018; Dixon and Barros, 2019; Vanholme et al., 2019). In addition, lignin functions as a conduit for the transport of water and nutrients through the plant vascular system (Liu et al., 2018; Dixon and Barros, 2019; Vanholme et al., 2019).

Lignin is a phenolic polymer synthesized through the phenylpropanoid pathway, which involves multiple enzymes and intermediates (Dixon et al., 2001; Vanholme et al., 2010; Barros et al., 2019a). The pathway begins with the conversion of the aromatic amino acid phenylalanine to cinnamic acid through the action of the enzyme phenylalanine ammonia-lyase (PAL) (Dixon and Paiva, 1995). Cinnamic acid is then converted into three major monomers, also known as monolignols, namely *p*-coumaryl alcohol, coniferyl alcohol, and sinapyl alcohol, through the concerted action of cinnamate 4-hydroxylase (C4H), 4-coumarate CoA ligase (4CL), hydroxycinnamoyl-CoA shikimate/quinate hydroxycinnamoyltransferase (HCT), p-coumaroyl shikimate/quinate 3-hydroxylase (C3H), caffeoyl-CoA 3-O-methyltransferase (CCoAOMT), ferulate 5-hydroxylase (F5H), caffeic acid/5-hydroxyferulic acid O-methyltransferase (COMT), cinnamoyl-CoA reductase (CCR), and cinnamyl alcohol dehydrogenase (CAD) (Boerjan et al., 2003; Hisano et al., 2009; Vanholme et al., 2010). These monolignols are transported to plant cell walls where they are oxidized by apoplastic peroxidases and laccases to form lignin polymers in different patterns (Vanholme et al., 2010; Barros et al., 2019a).

Coumarate 3-hydroxylase (C3H) belongs to a large family of cytochromes P450 and is one of the key enzymes in lignin biosynthesis (Reddy et al., 2005; Ralph et al., 2006; Ziebell et al., 2010; Tong et al., 2015). C3H uses p-coumaroyl shikimate as a substrate to direct the synthesis of guaiacyl and syringyl monolignols (Hisano et al., 2009; Ziebell et al., 2010; Ralph et al., 2012; Tong et al., 2015). Given its central role in the synthesis of S and G lignin, C3H has been targeted to reduce lignin content and alter composition in multiple plant species. Downregulation of *C3H* at the RNA level in alfalfa leads to reduced lignin content and altered composition with improved digestibility (Reddy et al., 2005; Ralph et al., 2006; Ziebell et al., 2010; Tong et al., 2015; Getachew et al., 2018). Downregulation of *C3H* in poplar and maize also leads to reduced lignin content and altered composition with improved saccharification efficiency for biofuel and pulping industries (Ralph et al., 2012; Fornalé et al., 2015).

Genome editing approaches, such as CRISPR/Cas9, have revolutionized the field of plant biotechnology through genome engineering, facilitating important trait-driven improvement in major crop species (Chen et al., 2020; Wolabu et al., 2020a, 2020b; Bottero et al., 2021; Zheng et al., 2021; Bao et al., 2022; Kumar et al., 2022; Nerkar et al., 2022; Singer et al., 2022; Wolabu et al., 2023). CIRSPR/Cas9 has been employed to manipulate lignin content in various plant species to improve the efficiency of lignocellulose processing and enhance forage quality, digestibility, and saccharification efficiency (Park et al., 2017; Jang et al., 2021; Afifi et al., 2022; Roy et al., 2023; Wolabu et al., 2023; Laksana et al., 2024). A recent study that altered flowering time via multiplex-CRISPR/Cas9 edition of a florigen in alfalfa generated promising lines with improved biomass yield and quality (Wolabu et al., 2023). Editing the switchgrass *4CL* gene has also been shown to generate mutant lines with reduced lignin content and improved sugar release (Park et al., 2017). Genome-editing has also been applied in wood species for lignin manipulation. By targeting *CAFFEOYL SHIKIMATE ESTERASE* (*CSE*) and *PHBMT1* in poplar, Zhao et al. (2021) and Jang et al. (2021) demonstrated that precise editing of targeted genes via CRISPR/Cas9 can improve plant lignocellulosic biomass production with less lignin. Recently, Sulis et al. (2023) reported the impact of an assembled multiplex CRISPR/Cas9 genome editing strategy by targeting six genes, namely *C3H*, *CCoAOMT*, *AldOMT*, *PAL*, *C4H*, and *CAD*, in poplar. They used different multigenic approaches to edit these genes to improve the chemical and physical properties of the woody plant and achieve optimized fiber production for the pulping industry. Edited lines exhibited varying degrees of loss-of-function mutations in the target genes with biallelic loss-of-function editing of all target genes (Sulis et al., 2023). Sugarcane lignin content reduction (up to 51% with high S/G ratio) was achieved using the CRISPR/Cas9 genome editing system by targeting SoLIM transcription factors that are involved in the lignin biosynthesis pathway (Laksana et al., 2024). In this regard, modern genome editing approaches offer great opportunities to reduce lignin content and/or to alter composition to improve product quality without compromising plant growth and resilience. This is one of the breeding goals to improve forage nutritional quality and fiber production (Park et al., 2017; Wolabu et al., 2020a; Nerkar et al., 2022; Roy et al., 2023; Sulis et al., 2023; Wolabu et al., 2023).

In this study, four specific target gRNAs were designed to knock out the alfalfa *C3H* (*MsC3H)* gene using a previously optimized multiplex CRISPR/Cas9 system to manipulate lignin content and composition for forage quality improvement. The generated tetra-allelic homozygous *Msc3h* mutant lines showed significantly reduced lignin content and a low level of ADF and NDF with no significant difference in biomass yield but with improved digestibility and nutritional values. These lines could be integrated into alfalfa breeding programs to generate elite transgene-free alfalfa cultivars with reduced lignin and improved forage quality.

## Materials and methods

### Plant materials and growth conditions

Alfalfa (*Medicago sativa* L.) cultivar, Regen-SY4D, was used for genome editing and genetic transformation. CRISPR/Cas9-edited lines and the non-edited control (empty vector-EV) were grown in greenhouse under conditions of 22°/19°C day/night temperature, 16/8 h day/night photoperiod, 150 µmol.m^-2^s^-1^ light intensity, and 70%-80% relative humidity. All alfalfa lines analyzed in this study were maintained and vegetatively propagated via stem cuttings.

### Multiplex gRNAs-CRISPR/Cas9 vector construction and plant transformation

To permanently mutate the alfalfa *C3H* (*MsC3H*) gene, we used a previously optimized efficient multiplex gRNAs-CRISPR/Cas9 system (Wolabu et al., 2020a) to generate tetra-allelic mutants in the complex alfalfa genome. To examine any potential genotypic SNPs that might influence the genome-editing efficiency due to mismatch among four alleles of *MsC3H*, the genomic DNA fragment of *MsC3H* spanning the target region was amplified from the Regen-SY4D genomic DNA by PCR and cloned into the pGEM-Teasy vector (Promega, Madison, WI, USA). At least twenty clones were randomly picked for Sanger sequencing to determine the sequence identity and presence of any SNPs in the conserved coding regions of four alfalfa alleles. Four *MsC3H* gRNAs, gRNA1-CCATACCCACTTCCCATCAT, gRNA2- ACAAAACTCTCAAACATCTA, gRNA3- TGAGGTTTTTGATATTGGTG, and gRNA4-TGGGGTATCATGGAAGAAGC upstream of respective PAM, AGG, TGG, AGG and TGG sites, were designed to target the first exon of the *MsC3H* gene using the web-based tool CRISPR-P (http://cbi.hzau.edu.cn/cgi-bin/CRISPR) (Lei et al., 2014). All designed gRNAs (spacers) were inserted between tRNA and gRNA scaffolds to be clustered in a tandem manner using the Golden Gate assembly method (Engler et al., 2008). The pGTR plasmid, which contains the tRNA-gRNA fragment, was used as a template to synthesize the polycistronic tRNA-gRNA (PTG) for construction of the multiplex tRNA-*MsC3H* spacer-gRNA (Xie et al., 2015; Wolabu et al., 2020a). First, the overlapping PCR products were purified using the Spin Column PCR Product Purification Kit (Promega, Madison, WI, USA) following the manufacturer’s instruction (www.promega.com). The PCR products containing four *MsC3H*-spacers were then inserted into the optimized vector with the AtU6-tRNA-*MsC3H*-gRNAs-AtUBQ10-Cas9-pRGEB31-bar backbone by digestion and ligation using Fok I (NEB) and BsaI (Wolabu et al., 2020a). Insertion of all multiplexed gRNAs into the CRISPR/Cas9 module was verified by DNA sequencing. The resulting binary vector, *MsC3H-gRNA-CRISPR/Cas9*, was transformed into the *Agrobacterium tumefaciens* strain, *EHA105*. *Agrobacterium*-mediated transformation of alfalfa was carried out following the protocol described earlier by Wolabu et al. (2020a) to generate CRIPR/Cas9-edited alfalfa *Msc3h* mutants.

### Genotyping of CRISPR*/*Cas9-edited *Msc3h* lines

To identify and select CRISPR/Cas9-edited *Msc3h* lines, multiple rigorous genotyping steps were applied. First, the transgenic lines were screened by PCR for presence or absence of the *BAR* gene using *BAR* gene specific primers (PPT-F + PPT-R). The *BAR* gene-positive transgenic lines were subjected to a second PCR amplification of the target region spanning the designed gRNAs with *MsC3H*-specific primers, followed by Sanger sequencing. The amplified PCR products were purified after treating with enzymes Antarctic phosphatase and exonuclease I (Wolabu et al., 2020a) and sequenced using the Sanger sequencing method to determine the mutation signature (double peak in the electropherogram upstream of PAM) in at least one of the gRNAs (gRNA1, gRNA2, gRNA3, and gRNA4). Then, transgenic lines considered as putative heterozygous and/or chimeric were selected and subjected to phenotypic analysis for desirable traits. Furthermore, promising lines with desirable phenotypes were characterized for homozygosity by cloning the target regions spanning the gRNAs (gRNA1, gRNA2, gRNA3, and gRNA4) into the pGEM-T Easy vector. Twenty colonies were randomly picked for each mutant line, and each plasmid DNA was sequenced using the Sanger sequencing method to determine the nature of mutation events in each *Msc3h* line (homozygous, heterozygous, or chimeric mutation) by aligning the reads with the reference sequence using the SeqMan Pro 15.0.1 (DNASTAR software for life scientists) (https://www.dnastar.com/quote-request/). All primer pairs used to genotype the plasmids and the transgenic lines in this study are listed in **Supplementary Table S2**.

### Tissue sampling and lignin composition analysis

For lignin analysis, stem samples were harvested at the early flower bud growth stage (the stage when 10% of the plants showed first flower buds). Pooled stem sections between node 4 and 7 from the bottom were collected and dried at a fixed temperature of 55° ± 5°C for 10 days followed by grinding into powder using a Wiley mill. The ground samples were extracted with chloroform/methanol (2:1, v/v), methanol, and water. Lignin composition was determined using a modified thioacidolysis method as described previously (Chen et al., 2021), with 4,4′-ethylidene bisphenols as the internal standard. The total lignin contents are represented by the sum of total thioacidolysis monomer yields. Three technical replicates were used in the first lignin analysis of independent transgenic lines on a single plant basis (a total of 55 Msc3h lines). Based on the first record of lignin analysis and phenotyping of desirable agronomic traits, three promising candidate lines, *Msc3h-013, Msc3h-121*, and *Msc3h*-*158*, were selected for further analysis of lignin composition using six biological replicates.

### Determination of forage nutritional quality

To determine the forage biomass yield and nutritional quality (digestibility and nutritional values) of three selected *Msc3h* lines, above-ground plant tissues (stems and leaves) were harvested when 10% of plants showed their first flower buds. Forage nutritional quality assessment was carried out at the Ag Services and Resources Core Facility (ASRC), Noble Research Institute, LLC. Sample grinding and processing were performed following procedures described by Gou et al. (2018). Forage nutritional quality was determined with the near infrared reflectance spectroscopy (NIRS) system using a Foss NIRS 6500 monochromator with a scanning range of 1100-2500 nm (Foss NIR System). The following forage nutritional quality parameters were measured: acid detergent fiber (ADF), neutral detergent fiber (NDF) in percentage of dry matter basis, total digestible nutrients (TDN), relative feed value (RFV), *in vitro* dry matter digestibility (IVTDMD), crude protein (CP) content, minerals including magnesium (Mg), potassium (K), calcium (Ca), and phosphorus (P) content.

### Statistical analysis

At least six plants for each independent line were used as biological replicates for statistical analyses. Data shown in graph bars represent the mean ± SD. The analyses were performed using the Microsoft Excel software and the asterisks indicate significant differences based on Student’s *t*-test (*** p<0.001, ** p<0.01, * p<0.05). The relative advantage (%) of the tested lines over the control (EV) was calculated as the performance of the tested line minus the control divided by the control and multiplied by 100 [(Line tested – EV)/EVx100 = %].

## Results

### Generation, screening, and analysis of CRISPR/Cas9-mediated *Msc3h* mutant lines

**Figure 1:**
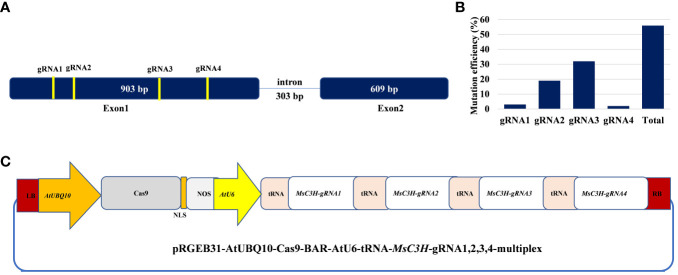
Schematic illustration of alfalfa *COUMARATE 3-HYDROXYLASE* (*MsC3H*) gene structure with designed multiplex gRNAs-CRISPR/Cas9 vector and genome editing efficiency in alfalfa. **(A)** *MsC3H* gene structure and four guide RNA sites with specific sequences in the coding region. **(B)** Mutation efficiency (%) of four guide RNAs at different target sites of *MsC3H*. **(C)** Illustration of the multiplex construct of *MsC3H*-gRNA1,2,3,4-CRISPR/Cas9 vector.

**Figure 2:**
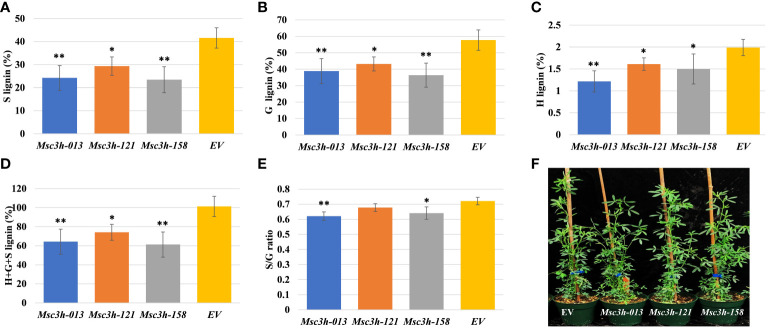
Analysis of lignin content and composition in stem tissues of three selected *Msc3h* mutant lines *Msc3h-013*, *Msc3h-121* and *Msc3h-158*. **(A)** S lignin content (%) in stem tissues of *Msc3h* lines and EV. **(B)** G lignin content (%) in stem tissues of *Msc3h* lines and EV. **(C)** H lignin content (%) in stem tissues of *Msc3h* lines and EV. **(D)** Total (H+G+S) lignin content (%) in stem tissues of *Msc3h* lines and EV. **(E)** S/G ratio in stem tissues of *Msc3h* lines and EV. **(F)** Phenotype of mutant lines *Msc3h-013*, *Msc3h-121*, and *Msc3h-158* with EV at early flower bud vegetative growth stage. Data represent mean values (± SD; n = 6). Statistics was analyzed using Student’s *t*-test (* p < 0.05; ** p < 0.01).

**Figure 3:**
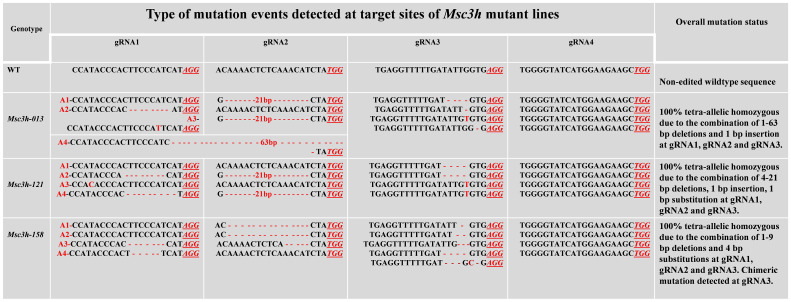
Molecular analysis of *MsC3H* mutation events (deletions/insertion/substitution) generated by multiplex gRNAs-CRISPR/Cas9 gene editing at four target sites of MsC3H. Mutation events (deletions, insertions, substitutions) of mutant lines *Msc3h-013*, *Msc3h-121*, and *Msc3h-158* occurred at each specific target site (gRNA1, gRNA2, gRNA3 and gRNA4) compared to wild type (WT). Deletions are indicated by red dashed lines; insertions or substitutions are indicated by red letters. PAMs are indicated by red underlined italicized letters. The four allelic copies are designated as red allele-1 (A1), allele-2 (A2), allele-3 (A3), and allele-4 (A4).

In this study, we mutated the *MsC3H* gene in alfalfa by employing a multiplex CRISPR/Cas9 system (Wolabu et al., 2020a). Four gRNAs were designed in the first exon of *MsC3H* at 111 bp (gRNA1), 156 bp (gRNA2), 465 bp (gRNA3), and 572 bp (gRNA4) downstream of the translation start codon, segments containing no SNPs across all four alleles of *MsC3H*, using CRISPR-P 2.0 (http://cbi.hzau.edu.cn/cgi-bin/CRISPR) (Lei et al., 2014) (**Figure 1A, C**). One hundred and thirty *BAR* (selection marker gene) positive lines were generated via *Agrobacterium*-mediated transformation. To identify CRISPR/Cas9-edited mutant lines, the target region spanning all four gRNAs were amplified from all 130 lines using a pair of primers flanking the target region (**Supplementary Table S2**). Of the 130 transgenic lines, 73 lines were identified as putative *Msc3h* mutant lines with at least one mutation signature (double peaks in sequencing chromatograph) at one of the target gRNA sites by Sanger sequencing of the PCR amplicons (**Figure 1B**). Of the 73 putative candidates, 55 lines were selected for lignin analysis based on phenotypic evaluation of desirable agronomic traits. Based on the results of lignin analysis, 12 candidate lines with reduced lignin content and altered composition were propagated for further characterization and statistical validation. Finally, three *Msc3h* mutant lines (*Msc3h-013*, *Msc3h-121*, and *Msc3h-158*) that exhibited similar growth patterns as control, empty vector (EV) plants (**Figure 2F**) were selected for analysis of the nature of mutation events at the target sites prior to a deeper analysis of lignin content and composition (**Figure 3**). The results showed that mutant line *Msc3h-013* had an 8-bp deletion and a 1-bp “T” insertion in Allele 2 (A2) and Allele 3 (A3), respectively, at gRNA1, and 21-bp deletions in A1 and A3 upstream of the PAM of gRNA2 (**Figure 3**). Moreover, a deletion of 63 bp between gRNA1 and gRNA2 in A4 had occurred, removing the entire sequence between gRNA1 and gRNA2 (**Figure 3**). Similarly, 1-4 bp deletions and 1-bp insertion upstream of PAM at gRNA3 were detected in four different alleles of *Msc3h-013* (**Figure 3**). Consequently, the mutation events at gRNA3 of *Msc3h-013* were sufficient to cause 100% tetra-allelic homozygous mutation and knock out the gene at a single site. Overall, the combined multiple mutation events occurred at gRNA1, gRNA2, and gRNA3 made line *Msc3h-013* a 100% tetra-allelic homozygous mutant (**Figure 3**, **Supplementary Figure S1A**). Similarly, analysis of mutant line Msc3h-121 showed 8- and 9-bp deletions in A2 and A4, respectively, along with a 1-bp “C to T” substitution in A1 at gRNA1 (**Figure 3**). *Msc3h-121* also showed 21-bp deletions (in A2 and A4) at gRNA2, 4-bp deletions (in A1 and A2) and a 1-bp “T” insertion (A3 and A4) at gRNA3 (**Figure 3**), making it a 100% tetra-allelic homozygous mutant. Therefore, the combined overall mutation events detected in *Msc3h-121* at three specific targets (gRNA1-3) led to a 100% tetra-allelic homozygous mutant (**Figure 3**, **Supplementary Figure S1B**). Diverse mutation events were also detected at gRNA1, gRNA2 and gRNA3 of the mutant line *Msc3h-158* (**Figure 3**, **Supplementary Figure S1C**). Mutation analysis indicated that 5-bp and 7-bp deletions (in A3 and A4) occurred at gRNA1 followed by 15-bp deletions (A1 and A2) and a 5-bp deletion (A3) at gRNA2 (**Figure 3**). At gRNA3, five different types of mutation events occurred across all four alleles of the *MsC3H* gene, including 1-bp deletion in A1, 2-bp deletion in A2, 3-bp deletion in A3, and 4-bp deletion in A4 (**Figure 3**, **Supplementary Figure S1C**), along with a 4-bp deletion and a 1-bp insertion in one of the alleles, indicating the chimeric nature of mutation events in *Msc3h-158*. Interestingly, the chimeric mutation also showed 100% homozygosity and the combined mutation events detected at gRNA1, gRNA2, and gRNA3 similarly produced 100% tetra-allelic homozygosity in the mutant line *Msc3h-158* (**Figure 3**, **Supplementary Figure S1C**). In summary, mutation event analysis indicated diverse mutation efficiencies and mutation events in gRNA1, gRNA2, and gRNA3 with complete mutations in all four alleles (copies) in the three selected mutant lines, *Msc3h-013*, *Msc3h-121*, and *Msc3h-158* (**Figure 3**, **Supplementary Figure S1A-C**). The overall mutation efficiency at gRNA3 was higher than that of any other gRNAs. The mutagenesis efficiency at gRNA3 was 32%, followed by 19% at gRNA2 and 4% at gRNA1, while gRNA4 had the lowest mutagenesis efficiency with only 2% (**Figure 1B**), which might be the reason why we did not detect mutations at gRNA4 in the selected three lines *Msc3h-013, Msc3h-121*, and *Msc3h-158* (**Figure 3**). All mutation events in these three lines occurred in the framework of designed gRNA1, 2, and 3 at 1-3 bp upstream of the PAM regions with a dominance of small deletions (1-9 bp). These results further highlight the usefulness of multiplex CRISPR/Cas9 system in maximizing the chances of obtaining knockout or edited mutants in the target genes through a single transformation event.

### Analysis of lignin content and composition in *Msc3h* mutant lines

To determine lignin content and composition (syringyl (S), guaiacyl (G), *p*-hydroxyl (H) units and total lignin) in all *Msc3h* mutants, we used stem samples consisting of internodes number 4, 5 and 6 counting from the bottom. Lignin deposition was determined in two phases by using the thioacidolysis method (Chen et al., 2021) for independent *Msc3h* mutant lines. In the first phase, 55 putative *Msc3h* mutant lines with various mutation events were analyzed to determine lignin content and composition compared to the non-edited EV control. The results showed a wide range of lignin composition, including the individual content of S, G, and H lignin units and the total lignin content. After performing lignin analysis on the 55 *Msc3h* lines, we selected 12 lines for vegetative propagation and further assessment of lignin content and composition and nutritional values (**Supplementary Figure S2A**). These twelve lines showed different agronomic traits with two of the lines having reduced growth while the others showed comparable plant height to the control (**Supplementary Figure S2A**). Finally, three of the 12 propagated lines (*Msc3h-013*, *Msc3h-121* and *Msc3h-158*), which were phenotypically comparable to EV and were tetra-allelic homozygous mutants, were selected and subjected to a second round of lignin content and composition analysis. Results showed that all three *Msc3h* lines had significantly reduced levels of S, G, H monomer yields, and total lignin compared to the EV control (**Figure 2A-D**). Remarkably, in the line *Msc3h-013*, the S, G, H monomer yields, and the total lignin content were reduced by 42%, 33%, 39%, and 36%, respectively, compared to EV (**Figure 2A-D**, **Supplementary Table S1**). Similarly, line *Msc3h-158* showed a reduction of 44%, 37%, 25% and 39% in S, G, H, and total lignin content, respectively, compared to the control. Line *Msc3h-121* showed a reduction of 29%, 25%, 20% and 27% in S, G, H, and total lignin content, respectively, compared to EV (**Figure 2A-D**, **Supplementary Table S1**). All three *Msc3h* mutant lines also showed 14%, 7% and 11% relative advantage of S/G ratio for *Msc3h-013, Msc3h-121* and *Msc3h-158*, respectively, over EV (**Figure 2E**, **Supplementary Table S1**). In conclusion, disruption of MsC3H substantially reduced key lignin components in selected lines compared to the control (**Figure 2A-E**, **Supplementary Table S1**).

### Analysis of forage digestibility and nutritional quality in *Msc3h* mutant lines

**Figure 4:**
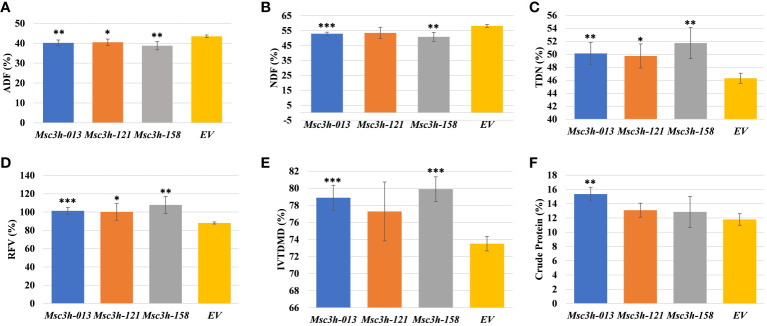
Analysis of forage quality in aboveground (leaves and stem) tissues of *Msc3h* mutant lines. **(A)** Acid Detergent Fiber (ADF) content (%) in *Msc3h* lines and EV. **(B)** Neutral Detergent Fiber (NDF) content (%) in *Msc3h* lines and EV. **(C)** Total Digestible Nutrients (TDN) content (%) in *Msc3h* lines and EV. **(D)** Relative Feed Value (RFV) (%) in *Msc3h* lines and EV. **(E)** *In Vitro* True Dry Matter Digestibility (IVTDMD) (%) in *Msc3h* lines and EV. **(F)** Crude protein content (%) in *Msc3h* lines and EV. Data represent mean values (± SD; n = 6) and were analyzed statistically using Student’s *t*-test (* p < 0.05; ** p < 0.01; *** p < 0.001).

**Figure 5:**
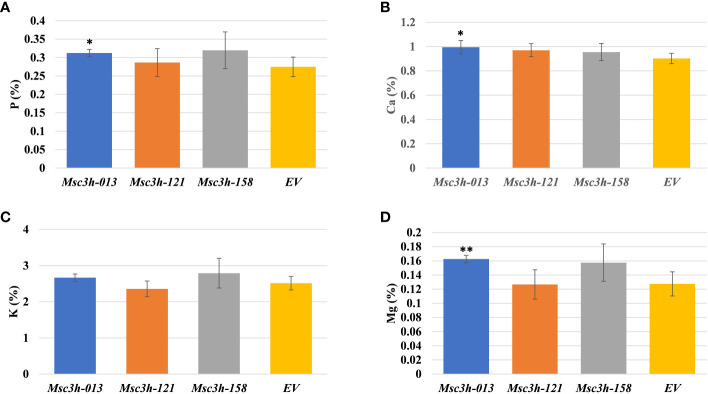
Analysis of forage macro-minerals contents in aboveground (leaves and stem) tissues of mutant lines *Msc3h-013*, *Msc3h-121* and *Msc3h-158* and EV. **(A)** Phosphorus (P) content (%). **(B)** Calcium (Ca) content (%). **(C)** Potassium (K) content (%). **(D)** Magnesium (Mg) content (%). Data represent mean values (± SD; n = 6) and were analyzed statistically using Student’s *t*-test (* p < 0.05; ** p < 0.01).

Given the significant reduction in lignin content in the selected *Msc3h* lines (*Msc3h-013*, *Msc3h-121*, and *Msc3h-158*), we evaluated the nutritional parameters of forage quality (ADF, NDF, TDN, RVF, IVTDMD, CP and macro-minerals) in whole shoot tissues of these lines at flower bud growth stage (**Figure 4A-F**, **5A-D**). The results showed that all three mutant lines had significantly lower levels of ADF with a relative advantage of 7-11% over EV (**Figure 4A**, **Supplementary Table S1**). Likewise, *Msc3h-013* and *Msc3h-158* also showed significantly reduced level of NDF with 9-13% relative advantage over EV (**Figure 4B**, **Supplementary Table S1**). Although not statistically significant, the NDF content in *Msc3h-121* was lower than that in EV with a relative advantage of 8%. In a similar trend, the nutritional values (specifically, TDN and RFV) in the three lines were significantly higher than that in EV (with 9-13% and 14-23% relative advantage over the EV for TDN and RFV, respectively) (**Figure 4C, D**, **Supplementary Table S1**). Highly significant (p < 0.001) elevation of IVTDMD was also recorded in mutant lines *Msc3h-013* and *Msc3h-158* with a relative advantage of 8% and 10% over EV, respectively (**Figure 4E**, **Supplementary Table S1**), followed by *Msc3h-121* with a relative advantage of 5% over EV, which was not statistically significant (**Figure 4E**, **Supplementary Table S1**). We also analyzed nutritional parameters such as crude protein (CP) and mineral contents in the three *Msc3h* mutant lines. Interestingly, *Msc3h-013* showed a significantly higher CP content with a relative advantage of 30% over EV (**Figure 4F**). Similarly, *Msc3h-013* showed a significantly high level of P, Ca and Mg content with a relative advantage of 15%, 11% and 27%, respectively, over EV (**Figure 5A, B, D**). Although not statistically significant, *Msc3h-121* and *Msc3h-158* also showed high levels of CP and four microminerals (P, Ca, K, and Mg) compared to EV (**Figure 4F**, **5A–D**). Overall, the mutant line *Msc3h-013* had the lowest lignin content, which was reflected by higher digestibility and nutritional values, followed by *Msc3h-158*, which ranked second based on lignin analysis (**Figure 2A-E**, **4A–E**). In conclusion, these results indicate that the multiplex CRISPR/Cas9-mediated gene editing system generated *Msc3h* mutant lines with significantly reduced lignin content and improved digestibility and nutritional quality.

Furthermore, we analyzed the correlation between lignin content/composition (S, G, H, and total lignin) and forage quality parameters, including digestibility (ADF and NDF) and nutritional values (TDN, RFV, IVTMDD and CP) to evaluate the importance of each variable (**Table 1** and **Supplementary Figure S3A-H**, **Figure S4A-H**, **Figure S5A–H**). Specifically, S lignin and total lignin showed a positive correlation with ADF with R^2^ = 0.52 and 0.50, respectively (**Supplementary Figure S3A, D**, **Table 1**), while S, G, and total lignin were positively correlated with NDF with R^2^ = 0.65, 0.62, 0.64, respectively (**Supplementary Figure S3E, F, H**, **Table 1**). However, the correlation between H lignin and ADF and NDF was consistently weaker than other lignin parameters (**Supplementary Figure S3C, G**, **Table 1**). On the other hand, S lignin was negatively correlated with TDN (R^2^ = 0.52), RFV (R^2^ = 0.62), IVTDMD (R^2^ = 0.75) and CP (R^2^ = 0.62) (**Supplementary Figure S4A, E**, **S5A, E**, **Table 1**). Similarly, G lignin and total lignin showed a moderate negative correlation with TDN, RFV, IVTDMD and CP (**Supplementary Figure S4B, D, F, H**, **S5B, D, F, H**, **Table 1**). Results of correlation analysis suggest that lignin content and composition directly alter forage nutrition quality. In summary, we generated Msc3h mutant lines with significantly reduced lignin content and improved digestibility and nutritional quality using the multiplex CRISPR/Cas9-mediated gene editing system.

**Table 1:** Correlation analyses of lignin composition *vs* forage quality traits in alfalfa *Msc3h* lines.

Correlation	(R^2^)	*p-*value	Status	Correlation (%)	(R^2^)	*p-*value	Status
ADF x S-lignin	0.52	0.01	Positive	TDN x S-lignin	0.52	0.01	Negative
ADF x G-lignin	0.47	0.01	Positive	TDN x G-lignin	0.47	0.01	Negative
ADF x H-lignin	0.28	0.07	Positive	TDN x H-lignin	0.28	0.07	Negative
ADF x Total-lignin	0.50	0.01	Positive	TDN x Total-lignin	0.50	0.001	Negative
NDF x S-lignin	0.65	0.001	Positive	RFV x S-lignin	0.62	0.02	Negative
NDF x G-lignin	0.62	0.001	Positive	RFV x G-lignin	0.58	0.04	Negative
NDF x H-lignin	0.40	0.05	Positive	RFV x H-lignin	0.36	0.05	Negative
NDF x Total-lignin	0.64	0.001	Positive	RFV x Total-lignin	0.61	0.002	Negative
IVTDMD x S-lignin	0.75	0.001	Negative	CP x S-lignin	0.65	0.001	Negative
IVTDMD x G-lignin	0.76	0.001	Negative	CP x G-lignin	0.60	0.002	Negative
IVTDMD x H-lignin	0.56	0.004	Negative	CP x H-lignin	0.64	0.001	Negative
IVTDMD x Total-lignin	0.76	0.001	Negative	CP x Total-lignin	0.59	0.003	Negative

## Discussion

Forage digestibility is negatively affected by lignin deposition in the feedstock (Baucher et al., 1999; Reddy et al., 2005). A number of studies targeting genes involved in lignin biosynthesis, such as *COMT*, *CAD*, *C4H*, *C3H*, *HCT*, *F5H*, and *CCoAMT*, via RNA interference or antisense approaches have shown the value of reducing lignin content in increasing forage digestibility of alfalfa plants (Marita et al., 2003; Reddy et al., 2005; Ralph et al., 2006; Shadle et al., 2007; Ziebell et al., 2010; Tong et al., 2015; Getachew et al., 2018; Singer et al., 2018; Barros et al., 2019b). However, transgenic approaches such as RNA interference, antisense, or artificial microRNA, which all manipulate gene functions at the RNA level and depend on sustained expression of transgenes in each generation, may suffer from transgene instability or silencing under field conditions, making these approaches less predictable (Jung and Altpeter, 2016). In this study, with the objective of overcoming these issues, we employed a multiplex CRISPR/Cas9 gene-editing approach to mutate the alfalfa *C3H* gene *(MsC3H)* at the DNA level and analyze the impact of altering lignin content and composition on plant growth and forage digestibility. In three *Msc3h* mutant lines, namely *Msc3h-013, Msc3h-121* and *Msc3h-158*, small Indels just upstream of the PAM regions of respective gRNAs were the most frequent mutations (**Figure 3**, **Supplementary Figure S1A-C**). Furthermore, large fragment deletions of 21-63 bp between gRNA1 and gRNA2 also occurred in the mutant line *Msc3h*-*158*, with 100% tetra-allelic homozygosity (**Figure 3**, **Supplementary Figure S1C**). Overall, analysis of editing events showed diverse mutation types in each specific target region with the highest mutagenesis efficiency in gRNA3 (**Figure 1B**). Consistent with these findings, similar mutation events with small Indels and some large, truncated fragments were observed in previous studies of alfalfa genome editing system targeting *MsPDS*, *MsPALM1*, *MsSGR*, *MsSPL8*, and *MsFTa1* genes (Chen et al., 2020; Wolabu et al., 2020a; Singer et al., 2022; Wolabu et al., 2023).

Remarkably*, Msc3h* mutant lines identified as tetra-allelic homozygous showed expected reduced lignin content and altered lignin composition in stem samples (**Figure 2A-D**). Interestingly, the results of thioacidolysis analysis showed wide ranges of lignin reduction in *Msc3h-013* and *Msc3h-158* followed by *Msc3h-121* with S lignin content lower than G lignin content (**Figure 2A, B**). Our results also showed a greatly reduced H lignin content, unlike previous reports on downregulation of the *C3H* gene using RNAi/antisense approaches (Chen and Dixon, 2007). The discrepancy of the H lignin contents between our study and the previous study could be attributed to the partial loss of function in the antisense/RNAi system and the complete loss of function in the gene-editing system. Overall, *Msc3h* mutant lines had significantly reduced lignin content compared to the control (**Figure 2A-D**, **Supplementary Table S1**). Furthermore, the three *Msc3h* mutant lines also exhibited reduced S/G ratio compared to EV plants. Previously, Reddy et al. (2005) reported the possibility of producing phenotypically normal alfalfa plants with strong downregulation in C3H activity and reduced lignification, which is in line with our *Msc3h* knockout results. The authors also suggested that there is no relationship between S/G ratios and digestibility, whereas the total lignin content is highly correlated with digestibility (Reddy et al., 2005). In summary, reduced components of lignin composition (monomeric units S, G, and H) were achieved in all three selected *Msc3h* mutant lines when compared to the EV control (**Figure 2A-E**, **Supplementary Table S1**).

The aim of this study was to reduce lignin content in alfalfa to improve forage digestibility and nutritional values by mutating the *MsC3H* gene. Small change in forage digestibility significantly influences animal performance efficiency (Shadle et al., 2007). The assessment of forage nutritional quality in the three *Msc3h* mutant lines (*Msc3h-013, Msc3h-121* and *Msc3h-158)* showed a significant impact of reducing lignin content in improving standard forage quality parameters (ADF, NDF, TDN, RVF, IVTDMD, CP and macro-minerals) (**Figure 4A-F**, **5A-D**). The three promising mutant lines consistently had lower levels of ADF and NDF and higher levels of TDN, RFV and IVTMDD compared to EV (**Figure 4A-E**). This is consistent with previous reports that decrease in ADF and NDF is associated with decrease in lignin content (Shadle et al., 2007; Getachew et al., 2018; Wolabu et al., 2023). Crude protein and mineral content were also significantly increased, thus enhancing forage quality. Among the three lines, *Msc3h-013* showed significantly higher crude protein (CP) content with a relative advantage of 30% over EV (**Figure 4F**) and significantly higher levels of Mg (27%), P (15%) and Ca (11%) relative to EV (**Figure 5A, B, D**). Overall, the mutant line *Msc3h-013* exhibited the lowest lignin content with superior improvement in digestibility and nutritional values, but slightly lower dry matter yield (**Supplementary Figure S2B**). *Msc3h-121* and *Msc3h-158* showed desirable agronomic traits comparable to the control (EV) without a significant effect on dry matter (**Supplementary Figure S2B**). Reduced biomass yield has been reported when *C3H* expression is strongly downregulated in transgenic alfalfa lines (Chen and Dixon, 2007). Furthermore, Reddy et al. (2005) targeted three specific cytochrome P450 enzymes involved in lignin pathway and generated transgenic lines with different lignin content and composition in alfalfa, suggesting the importance of fine-tuning the level of *C3H* or *C4H* expression to eliminate the possibility of reduced growth. In a similar manner, Chen and Dixon (2007) used alfalfa transgenic lines expressing antisense constructs for downregulation of six genes (including *MsC3H*) to determine the relationship between lignin content/composition and chemical/enzymatic saccharification. However, our study targeted a single gene (*MsC3H*) for forage quality improvement. The difference in the results obtained in this study and those previously reported in relation to the content and composition of lignin and agronomic parameters of nutritional quality could be attributed to the technology used, the germplasm, environmental conditions, and/or developmental factors that influence the complex biosynthesis of monolignols. However, as a trade-off for the reduction in dry matter, *Msc3h-013* showed superior performance in digestibility that substantially benefits nutritional values, CP, and mineral contents. This line could be used as a valuable genetic resource in alfalfa breeding programs. Decreases in ADF and NDF are associated with decreases in lignin components (Shadle et al., 2007; Getachew et al., 2018; Wolabu et al., 2023). Recently, we employed the CRISPR/Cas9 system to target the *MsFTa1* gene in alfalfa to delay flowering and found that the resulting late flowering *Msfta1* mutant lines produced not only greater biomass, as expected, but also had reduced lignin and fiber content (Wolabu et al., 2023). Such findings suggest that it is possible to design efficient gRNAs to knock out multiple genes with combined developmental traits, such as delayed flowering, delayed senescence, and reduced lignin traits to improve biomass yield and nutritional quality in alfalfa (Wolabu et al., 2020a, 2023).

In conclusion, we generated *Msc3h* mutant lines with significantly reduced lignin and enhanced forage digestibility and nutritional quality using the multiplex CRISPR/Cas9-mediated gene editing system. These promising *Msc3h* mutant lines could be useful genetic resources in alfalfa breeding programs for the development of transgene-free mutant lines and germplasm enrichment.

## Data availability statement

The original contributions presented in the study are included in the article/**Supplementary Material**. Further inquiries can be directed to the corresponding author.

## Author contributions

TW: Formal Analysis, Investigation, Writing – original draft. KM: Formal Analysis, Writing – original draft. FC: Investigation, Writing – review & editing. IT: Writing – review & editing, Data curation. MU: Writing – review & editing, Conceptualization. MT: Writing – review & editing. LC: Writing – review & editing, Investigation. ZW: Conceptualization, Writing – review & editing. JW: Data curation, Project administration, Supervision, Writing – review & editing.

## Supplementary material

Supplementary Figure 1 Molecular analysis of mutation events in lines *Msc3h-013*, *Msc3h-121*, and *Msc3h-158*. **(A)** Mutation events occurred in *Msc3h-013* at gRNA3 with one to four nucleotide deletions and one nucleotide insertion. **(B)** Mutation events occurred in *Msc3h-121* at gRNA3 with one to four nucleotide deletions and one nucleotide insertion. **(C)** Mutation events occurred in *Msc3h-158* at gRNA3 with one to four nucleotide deletions and one nucleotide substitution. All three promising *Msc3h* lines showed tetra-allelic homozygosity at gRNA3, generating 100% tetra-allelic homozygous mutations in *MsC3H*. Red arrows show PAM (protospacer adjacent motif) sequences.

Supplementary Figure 2
**(A)** Phenotype of twelve selected *Msc3h* mutant lines at flower bud vegetative stages showing different agronomic traits as compared to EV. Tetra-allelic homozygous lines with reduced lignin content and composition were propagated for replicated statistical analysis. **(B)** Analysis of dry weight (g) of three selected promising candidate *Msc3h* mutant lines *vs* EV.

Supplementary Figure 3 Correlation analysis of lignin composition of stems with forage quality traits in *Msc3h* mutant lines. **(A)** S lignin (%) x ADF (%). **(B)** G lignin (%) x ADF (%). **(C)** H lignin (%) x ADF (%). (**D)** H+G+S lignin (%) x ADF (%). **(E)** S lignin (%) x NDF (%). **(F)** G lignin (%) x NDF (%). **(G)** H lignin (%) x NDF (%). **(H)** H+G+S lignin (%) x NDF (%).

Supplementary Figure 4 Correlation analysis of lignin composition of stems with forage quality traits in *Msc3h* mutant lines. **(A)** S lignin (%) x TDN (%). **(B)** G lignin (%) x TDN (%). **(C)** H lignin (%) x TDN (%). **(D)** H+G+S lignin (%) x TDN (%). **(E)** S lignin (%) x RFV (%). **(F)** G lignin (%) x RFV (%). **(G)** H lignin (%) x RFV (%). **(H)** H+G+S lignin (%) x RFV (%).

Supplementary Figure 5 Correlation analysis of lignin composition of stems with forage quality traits in *Msc3h* mutant lines. **(A)** S lignin (%) x IVTDMD (%). **(B)** G lignin (%) x IVTDMD (%). **(C)** H lignin (%) x IVTDMD (%). **(D)** H+G+S lignin (%) x IVTDMD (%). **(E)** S lignin (%) x CP (%). **(F)** G lignin (%) x CP (%). **(G)** H lignin (%) x CP (%). **(H)** H+G+S lignin (%) x CP (%).

Supplementary Table 1 Relative advantage (%) of lignin and nutrition quality in alfalfa *Msc3h* mutant lines over EV (control).

Supplementary Table 2 Sequences of primers used in this study.

## References

[B1] Afifi O. A. Tobimatsu Y. Lam P. Y. Martin A. F. Miyamoto T. Osakabe Y.. (2022). Genome-edited rice deficient in two 4-coumarate:coenzyme A ligase genes displays diverse lignin alterations. Plant Physiol. 190, 2155–2172. doi: 10.1093/plphys/kiac450 36149320 PMC9706450

[B2] Bao Q. Wolabu T. W. Zhang Q. Zhang T. Liu Z. Sun J.. (2022). Application of CRISPR/Cas9 technology in forages. Grassland Res. 4, 244–251. doi: 10.1002/glr2.12036

[B3] Barros J. Escamilla-Trevino L. Song L. Rao X. Serrani-Yarce J. C. Palacios M. D.. (2019a). 4-Coumarate 3-hydroxylase in the lignin biosynthesis pathway is a cytosolic ascorbate peroxidase. Nat. Commun. 10, 1994. doi: 10.1038/s41467-019-10082-7 31040279 PMC6491607

[B4] Barros J. Temple S. Dixon R. A. (2019b). Development and commercialization of reduced lignin alfalfa. Curr. Opin. Biotechnol. 56, 48–54. doi: 10.1016/j.copbio.2018.09.003 30268938

[B5] Baucher M. Bernard-Vailhé M. A. Chabbert B. Besle J. M. Opsomer C. Van Montagu M.. (1999). Down-regulation of cinnamyl alcohol dehydrogenase in transgenic alfalfa (Medicago sativa L.) and the effect on lignin composition and digestibility. Plant Mol. Biol. 39, 437–447. doi: 10.1023/A:1006182925584 10092173

[B6] Bhuiyan N. H. Selvaraj G. Wei Y. King J. (2009). Role of lignification in plant defense. Plant Signal Behav. 4, 158–159. doi: 10.4161/psb.4.2.7688 19649200 PMC2637510

[B7] Boerjan W. Ralph J. Baucher M. (2003). Lignin biosynthesis. Ann. Rev. Plant Biol. 54, 519–546. doi: 10.1146/annurev.arplant.54.031902.134938 14503002

[B8] Bottero E. Massa G. González M. Stritzler M. Tajima H. Gómez C.. (2021). Efficient CRISPR/Cas9 genome editing in alfalfa using a public germplasm. Front. Agron. 3. doi: 10.3389/fagro.2021.661526

[B9] Capstaff N. M. Miller A. J. (2018). Improving the yield and nutritional quality of forage crops. Front. Plant Sci. 9. doi: 10.3389/fpls.2018.00535 PMC5928394 29740468

[B10] Chen F. Dixon R. A. (2007). Lignin modification improves fermentable sugar yields for biofuel production. Nat. Biotechnol. 25, 759–761. doi: 10.1038/nbt1316 17572667

[B11] Chen H. Zeng Y. Yang Y. Huang L. Tang B. Zhang H.. (2020). Allele-aware chromosome-level genome assembly and efficient transgene-free genome editing for the autotetraploid cultivated alfalfa. Nat. Commun. 11, 2494. doi: 10.1038/s41467-020-16338-x 32427850 PMC7237683

[B12] Chen F. Zhuo C. Xiao X. Pendergast T. H. Devos K. M. (2021). A rapid thioacidolysis method for biomass lignin composition and tricin analysis. Biotechnol. Biofuels 14, 18. doi: 10.1186/s13068-020-01865-y 33430954 PMC7798261

[B13] Dixon R. A. Barros J. (2019). Lignin biosynthesis: old roads revisited and new roads explored. Open Biol. 9, 190215. doi: 10.1098/rsob.190215 31795915 PMC6936255

[B14] Dixon R. A. Chen F. Guo D. Parvathi K. (2001). The biosynthesis of monolignols: a ‘‘metabolic grid’’ or independent pathways to guaiacyl and syringyl units? Phytochemistry 57, 1069–1084. doi: 10.1016/s0031-9422(01)00092-2 11430980

[B15] Dixon R. A. Paiva N. L. (1995). Stress-induced phenylpropanoid metabolism. Plant Cell 7, 1085–1097. doi: 10.2307/3870059 12242399 PMC160915

[B16] Engler C. Kandzia R. Marillonnet S. (2008). A one pot, one step, precision cloning method with high throughput capability. PloS One 3, e3647. doi: 10.1371/journal.pone.0003647 18985154 PMC2574415

[B17] Fornalé S. Rencoret J. Garcia-Calvo L. Capellades M. Encina A. Santiago R.. (2015). Cell wall modifications triggered by the down-regulation of Coumarate 3-hydroxylase-1 in maize. Plant Sci. 236, 272–282. doi: 10.1016/j.plantsci.2015.04.007 26025540

[B18] Gallego-Giraldo L. Bhattarai K. Pislariu C. I. Nakashima J. Jikumaru Y. Kamiya Y.. (2014). Lignin modification leads to increased nodule numbers in alfalfa. Plant Physiol. 164, 1139–1150. doi: 10.1104/pp.113.232421 24406794 PMC3938609

[B19] Getachew G. Laca E. A. Putnam D. H. Witte D. McCaslin M. Ortega K. P.. (2018). The impact of lignin downregulation on alfalfa yield, chemical composition, and in *vitro* gas production. J. Sci. Food Agric. 98, 4205–4215. doi: 10.1002/jsfa.8942 29406620

[B20] Gou J. Debnath S. Sun L. Flanagan A. Tang Y. Jiang Q.. (2018). From model to crop: functional characterization of SPL8 in M. truncatula led to genetic improvement of biomass yield and abiotic stress tolerance in alfalfa. Plant Biotechnol. J. 16, 951–962. doi: 10.1111/pbi.12841 28941083 PMC5866946

[B21] Hisano H. Nandakumar R. Wang Z. Y. (2009). Genetic modification of lignin biosynthesis for improved biofuel production. Vitro Cell. Dev. Biol. Plant. 45, 306–313. doi: 10.1007/s11627-009-9219-5

[B22] Jang H. A. Bae E. K. Kim M. H. Park S. J. Choi N. Y. Pyo S. W.. (2021). CRISPR-knockout of CSE gene improves saccharification efficiency by reducing lignin content in hybrid poplar. Int. J. Mol. Sci. 22, 9750. doi: 10.3390/ijms22189750 34575913 PMC8466951

[B23] Jung J. H. Altpeter F. (2016). TALEN mediated targeted mutagenesis of the caffeic acid O-methyltransferase in highly polyploid sugarcane improves cell wall composition for production of bioethanol. Plant Mol. Biol. 92, 131–142. doi: 10.1007/s11103-016-0499-y 27306903 PMC4999463

[B24] Kumar D. Yadav A. Ahmad R. Dwivedi U. N. Yadav K. (2022). CRISPR-based genome editing for nutrient enrichment in crops: a promising approach toward global food security. Front. Genet. 13. doi: 10.3389/fgene.2022.932859 PMC9329789 35910203

[B25] Lacefield D. G. (2004). “Alfalfa quality: What it is? What can we do about it? And will it pay?,” in Proceedings, National Alfalfa Symposium, San Diego, CA: UC Cooperative Extension, University of California, Davis, 13-15 December 2004.

[B26] Laksana C. Sophiphun O. Chanprame S. (2024). Lignin reduction in sugarcane by performing CRISPR/Cas9 site-direct mutation of SoLIM transcription factor. Plant Sci. 111987 (340), 1–12. doi: 10.1016/j.plantsci.2024.111987 38220093

[B27] Lei Y. Lu L. Liu H. Y. Li S. Xing F. Chen L. L. (2014). CRISPR-P: a web tool for synthetic single-guide RNA design of CRISPR-system in plants. Mol. Plant 7, 1494–1496. doi: 10.1093/mp/ssu044 24719468

[B28] Li X. Weng J. K. Chapple C. (2008). Improvement of biomass through lignin modification. Plant J. 54, 569–581. doi: 10.1111/j.1365-313X.2008.03457.x 18476864

[B29] Liu Q. Luo L. Zheng L. (2018). Lignins: biosynthesis and biological functions in plants. Int. J. Mol. Sci. 19, 335. doi: 10.3390/ijms19020335 29364145 PMC5855557

[B30] Marita J. M. Ralph J. Hatfield R. D. Guo D. Chen F. Dixon R. A. (2003). Structural and compositional modifications in lignin of transgenic alfalfa down-regulated in caffeic acid 3-O-methyltransferase and caffeoyl coenzyme A 3-O-methyltransferase. Phytochemistry 62, 53–65. doi: 10.1016/S0031-9422(02)00434-X 12475619

[B31] Nerkar G. Devarumath S. Purankar M. Kumar A. Valarmathi R. Devarumath R.. (2022). Advances in crop breeding through precision genome editing. Front. Genet. 13. doi: 10.3389/fgene.2022.880195 PMC9329802 35910205

[B32] Park J. J. Yoo C. G. Flanagan A. Pu Y. Debnath S. Ge Y.. (2017). Defined tetra-allelic gene disruption of the 4-coumarate: coenzyme A ligase 1 (Pv4CL1) gene by CRISPR/Cas9 in switchgrass results in lignin reduction and improved sugar release. Biotechnol. Biofuels 10, 284. doi: 10.1186/s13068-017-0972-0 29213323 PMC5708096

[B33] Ralph J. Akiyama T. Kim H. Lu F. Schatz P. F. Marita J. M.. (2006). Effects of coumarate 3-hydroxylase down-regulation on lignin structure. J. Biol. Chem. 281, 8843–8853. doi: 10.1074/jbc.M511598200 16421107

[B34] Ralph J. Akiyamam T. Coleman H. D. Mansfield S. D. (2012). Effects on lignin structure of coumarate 3-hydroxylase downregulation in poplar. Bioenergy Res. 5, 1009–1019. doi: 10.1007/s12155-012-9218-y 26366246 PMC4560085

[B35] Reddy M. S. Chen F. Shadle G. Jackson L. Aljoe H. Dixon R. A. (2005). Targeted down-regulation of cytochrome P450 enzymes for forage quality improvement in alfalfa (Medicago sativa L.). Proc. Natl. Acad. Sci. U.S.A. 102, 16573–16578. doi: 10.1073/pnas.0505749102 16263933 PMC1283808

[B36] Roy S. Manna S. Chowdhury S. Choudhury L. (2023). Improvement of large-scale production of lignocellulosic bioethanol through synthetic biology approaches: a comprehensive review. World J. Biol. Pharm. Health Sci. (WJBPHS) 14, 316–331. doi: 10./wjbphs.2023.14.3.0275

[B37] Shadle G. Chen F. Srinivasa Reddy M. S. Jackson L. Nakashima J. Dixon R. A. (2007). Down-regulation of hydroxycinnamoyl CoA: shikimate hydroxycinnamoyl transferase in transgenic alfalfa affects lignification, development and forage quality. Phytochemistry 68, 1521–1529. doi: 10.1016/j.phytochem.2007.03.022 17466347

[B38] Singer S. D. Burton H. K. Subedi U. Dhariwal G. K. Kader K. Acharya S.. (2022). The CRISPR/cas9- mediated modulation of SQUAMOSA PROMOTER-BINDING PROTEIN-LIKE 8 in alfalfa leads to distinct phenotypic outcomes. Front. Plant Sci. 12. doi: 10.3389/fpls.2021.774146 PMC8793889 35095953

[B39] Singer S. D. Hannoufa A. Acharya S. (2018). Molecular improvement of alfalfa for enhanced productivity and adaptability in a changing environment. Plant Cell Environ. 41, 1955–1971. doi: 10.1111/pce.13090 29044610

[B40] Sulis D. B. Jiang X. Yang C. Marques B. M. Matthews M. L. Miller Z.. (2023). Multiplex CRISPR editing of wood for sustainable fiber production. Science 381, 216–221. doi: 10.1126/science.add4514 37440632 PMC10542590

[B41] Tong Z. Li H. Zhang R. Ma L. Dong J. Wang T. (2015). Co-downregulation of the hydroxycinnamoyl-CoA:shikimate hydroxycinnamoyl transferase and coumarate 3-hydroxylase significantly increases cellulose content in transgenic alfalfa (Medicago sativa L.). Plant Sci. 239, 230–237. doi: 10.1016/j.plantsci.2015.08.005 26398807

[B42] Vanholme R. Demedts B. Morreel K. Ralph J. Boerjan W. (2010). Lignin biosynthesis and structure. Plant Physiol. 153, 895–905. doi: 10.1104/pp.110.155119 20472751 PMC2899938

[B43] Vanholme R. De Meester B. Ralph J. Boerjan W. (2019). Lignin biosynthesis and its integration into metabolism. Curr. Opin. Biotechnol. 56, 230–239. doi: 10.1016/j.copbio.2019.02.018 30913460

[B44] Wolabu T. W. Cong L. Park J. J. Bao Q. Chen M. Sun J.. (2020a). Development of a highly efficient multiplex genome editing system in outcrossing tetraploid alfalfa (*Medicago sativa*). Front. Plant Sci. 11. doi: 10.3389/fpls.2020.01063 PMC7380066 32765553

[B45] Wolabu T. W. Mahmood K. Jerez I. T. Cong L. Yun J. Udvardi M.. (2023). Multiplex CRISPR/Cas9- mediated mutagenesis of alfalfa FLOWERING LOCUS Ta1 (MsFTa1) leads to delayed flowering time with improved forage biomass yield and quality. Plant Biotechnol. J. 21, 1383–1392. doi: 10.1111/pbi.14042 36964962 PMC10281603

[B46] Wolabu T. W. Park J. J. Chen M. Cong L. Ge Y. Jiang Q.. (2020b). Improving the genome editing efficiency of CRISPR/Cas9 in Arabidopsis and Medicago truncatula. Planta 252, 15. doi: 10.1007/s00425-020-03415-0 32642859 PMC7343739

[B47] Xie K. Minkenberg B. Yang Y. (2015). Boosting CRISPR/Cas9 multiplex editing capability with the endogenous tRNA-processing system. Proc. Natl. Acad. Sci. U.S.A. 112, 3570–3575. doi: 10.1073/pnas.1420294112 25733849 PMC4371917

[B48] Zhao Y. Yu X. Lam P. Y. Zhang K. Tobimatsu Y. Liu C. J. (2021). Monolignol acyltransferase for lignin p-hydroxybenzoylation in Populus. Nat. Plants 7, 1288–1300. doi: 10.1038/s41477-021-00975-1 34354261

[B49] Zheng L. Wen J. Liu J. Meng X. Liu P. Cao N.. (2021). From model to alfalfa: Gene editing to obtain semidwarf and prostrate growth habits. Crop J. 10, 932–941. doi: 10.1016/j.cj.2021.11.008

[B50] Zhou C. Han L. Pislariu C. Nakashima J. Fu C. Jiang Q.. (2011). From model to crop: functional analysis of a STAY-GREEN gene in the model legume *Medicago truncatula* and effective use of the gene for alfalfa improvement. Plant Physiol. 157, 1483–1496. doi: 10.1104/pp.111.185140 21957014 PMC3252161

[B51] Ziebell A. Gracom K. Katahira R. Chen F. Pu Y. Ragauskas A.. (2010). Increase in 4-coumaryl alcohol units during lignification in alfalfa (Medicago sativa) alters the extractability and molecular weight of lignin. J. Biol. Chem. 285, 38961–38968. doi: 10.1074/jbc.M110.137315 20921228 PMC2998124

