# Supplementary figures and images for: Mutating alfalfa COUMARATE 3-HYDROXYLASE using multiplex CRISPR/Cas9 leads to reduced lignin deposition and improved forage quality
Source: Front Plant Sci. 2024 Mar 5;15:1363182. doi: 10.3389/fpls.2024.1363182 (PMC10948404; doi:10.3389/fpls.2024.1363182)

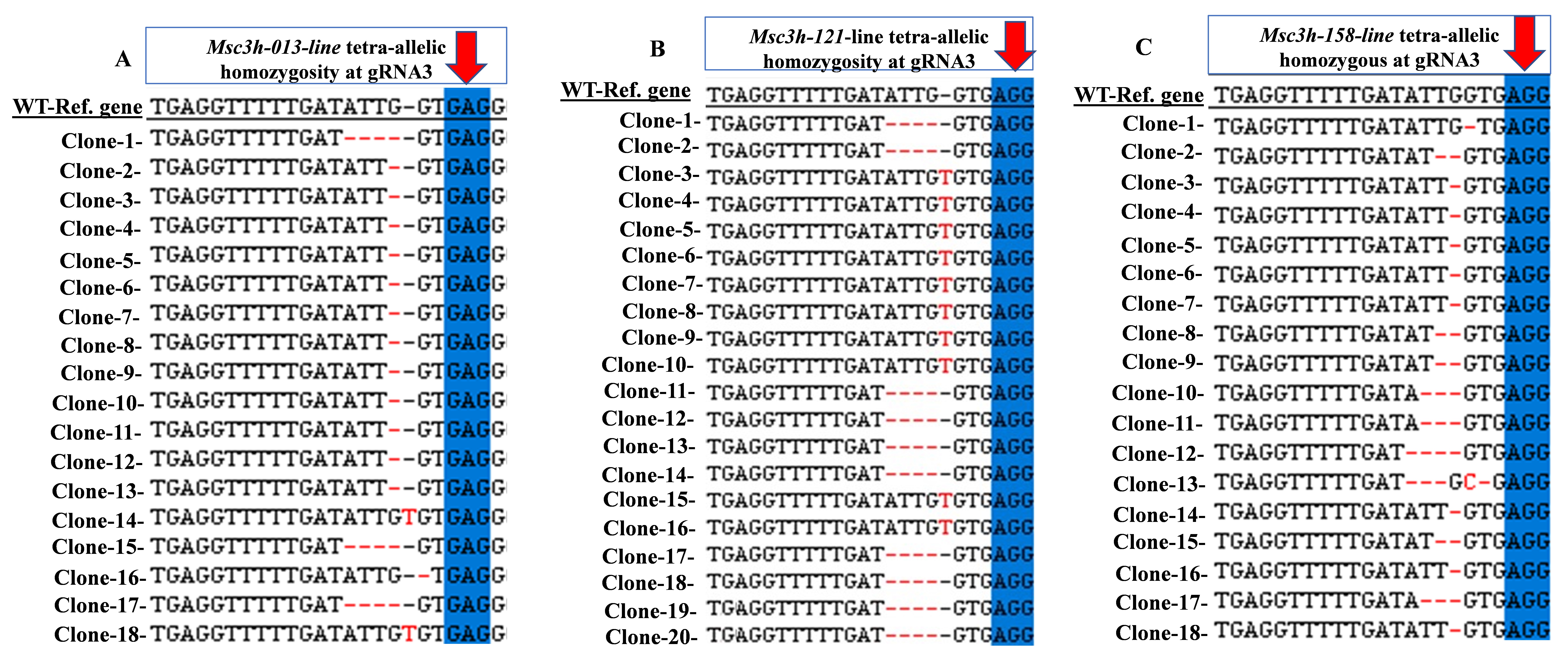

Supplement: Supplementary Figure 1 — Molecular analysis of mutation events in lines Msc3h-013, Msc3h-121, and Msc3h-158. (A) Mutation events occurred in Msc3h-013 at gRNA3 with one to four nucleotide deletions and one nucleotide insertion. (B) Mutation events occurred in Msc3h-121 at gRNA3 with one to four nucleotide deletions and one nucleotide insertion. (C) Mutation events occurred in Msc3h-158 at gRNA3 with one to four nucleotide deletions and one nucleotide substitution. All three promising Msc3h lines showed tetra-allelic homozygosity at gRNA3, generating 100% tetra-allelic homozygous mutations in MsC3H. Red arrows show PAM (protospacer adjacent motif) sequences. [file Image_1.jpeg]

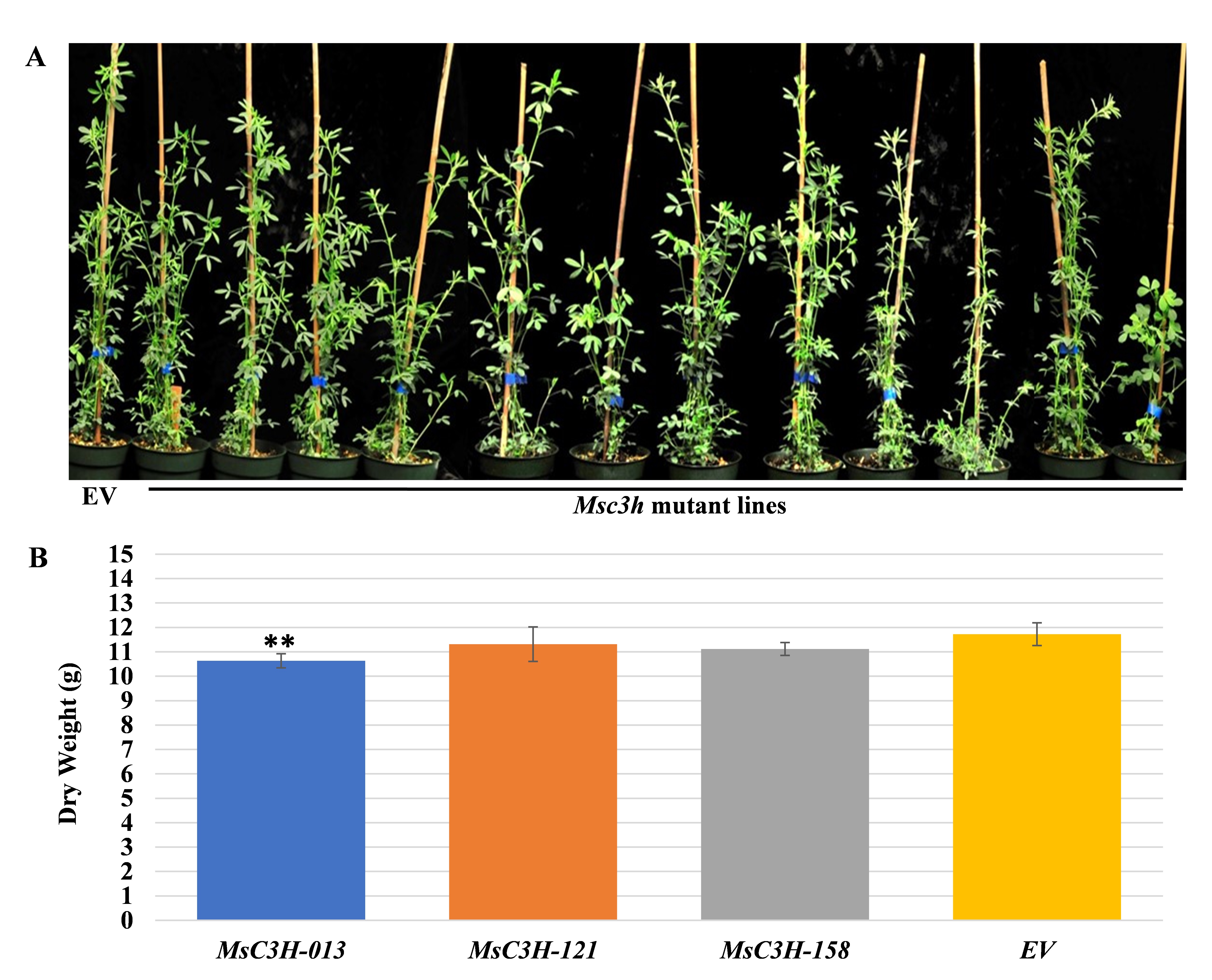

Supplement: Supplementary Figure 2 — (A) Phenotype of twelve selected Msc3h mutant lines at flower bud vegetative stages showing different agronomic traits as compared to EV. Tetra-allelic homozygous lines with reduced lignin content and composition were propagated for replicated statistical analysis. (B) Analysis of dry weight (g) of three selected promising candidate Msc3h mutant lines vs EV. [file Image_2.jpeg]

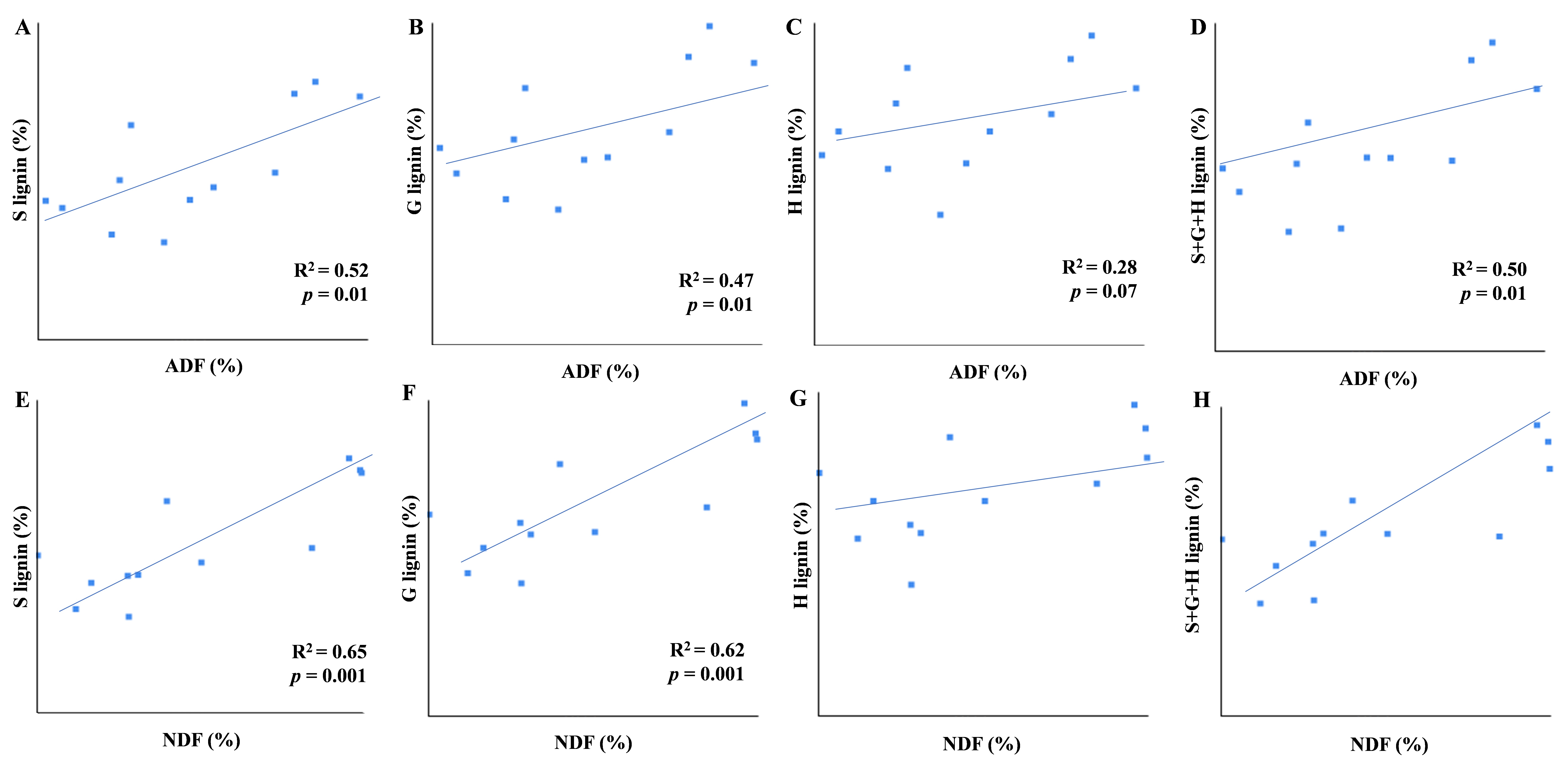

Supplement: Supplementary Figure 3 — Correlation analysis of lignin composition of stems with forage quality traits in Msc3h mutant lines. (A) S lignin (%) x ADF (%). (B) G lignin (%) x ADF (%). (C) H lignin (%) x ADF (%). (D) H+G+S lignin (%) x ADF (%). (E) S lignin (%) x NDF (%). (F) G lignin (%) x NDF (%). (G) H lignin (%) x NDF (%). (H) H+G+S lignin (%) x NDF (%). [file Image_3.jpeg]

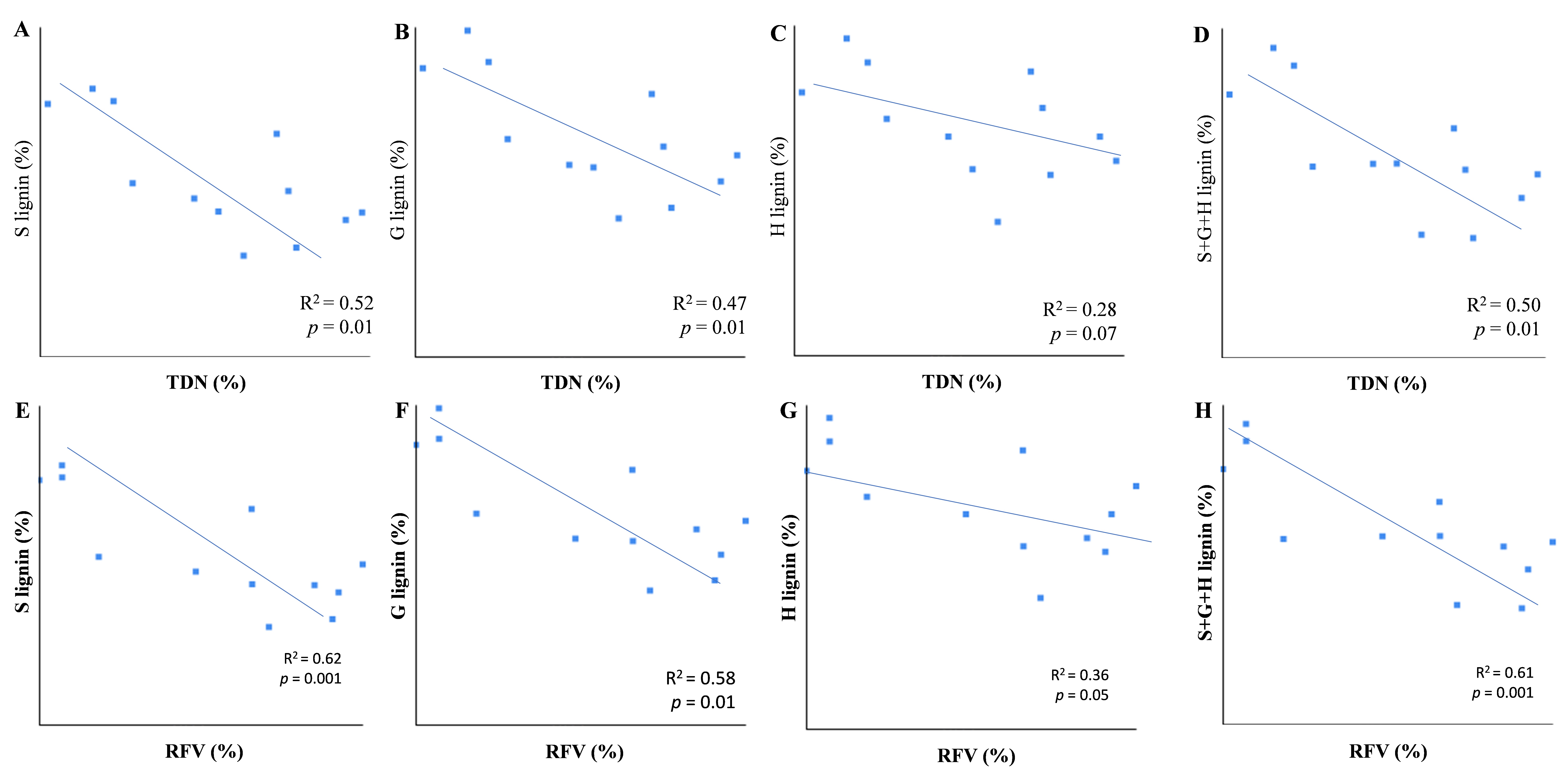

Supplement: Supplementary Figure 4 — Correlation analysis of lignin composition of stems with forage quality traits in Msc3h mutant lines. (A) S lignin (%) x TDN (%). (B) G lignin (%) x TDN (%). (C) H lignin (%) x TDN (%). (D) H+G+S lignin (%) x TDN (%). (E) S lignin (%) x RFV (%). (F) G lignin (%) x RFV (%). (G) H lignin (%) x RFV (%). (H) H+G+S lignin (%) x RFV (%). [file Image_4.jpeg]

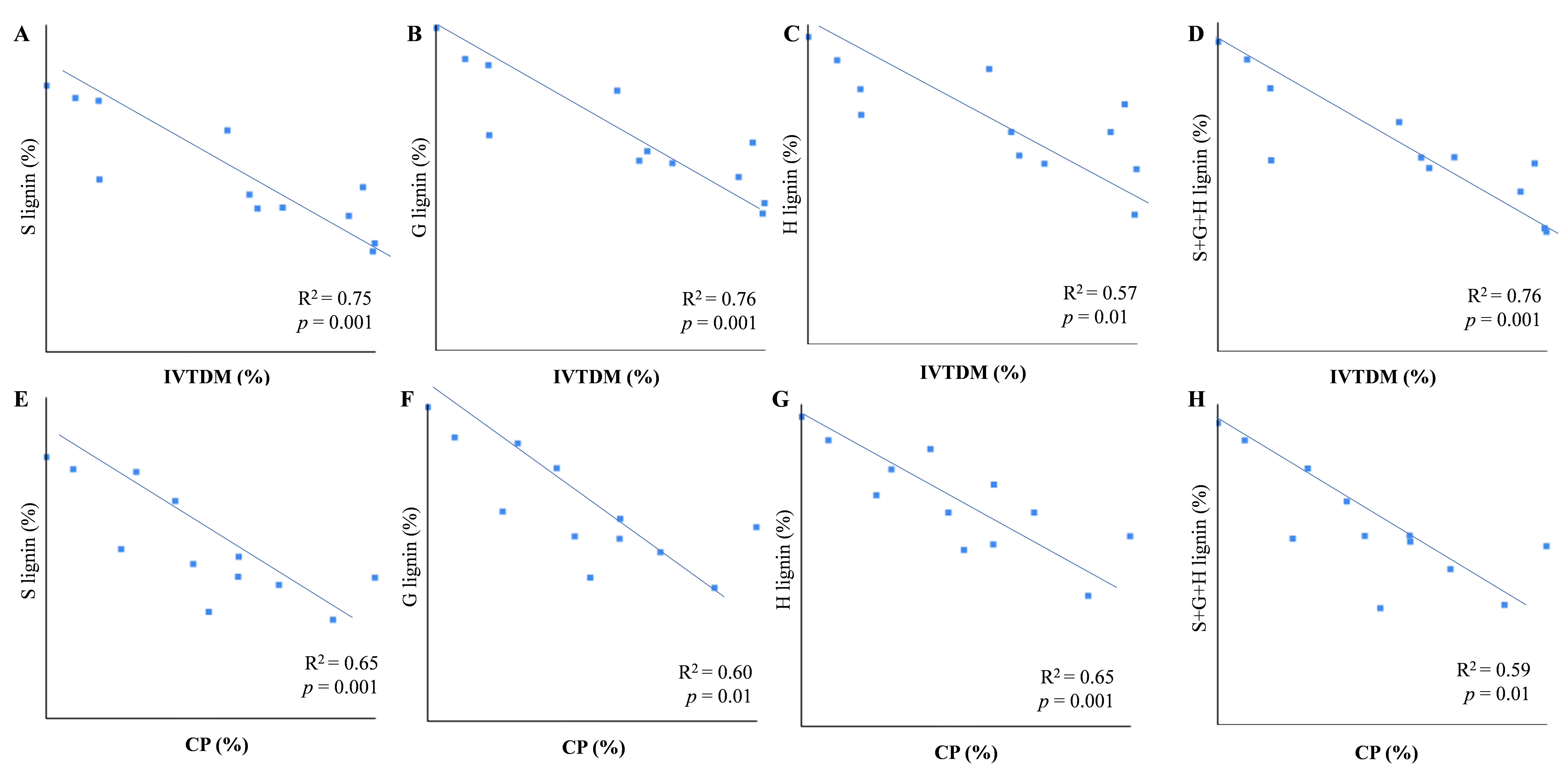

Supplement: Supplementary Figure 5 — Correlation analysis of lignin composition of stems with forage quality traits in Msc3h mutant lines. (A) S lignin (%) x IVTDMD (%). (B) G lignin (%) x IVTDMD (%). (C) H lignin (%) x IVTDMD (%). (D) H+G+S lignin (%) x IVTDMD (%). (E) S lignin (%) x CP (%). (F) G lignin (%) x CP (%). (G) H lignin (%) x CP (%). (H) H+G+S lignin (%) x CP (%). [file Image_5.jpeg]
