# Supplementary material for: Mutating alfalfa COUMARATE 3-HYDROXYLASE using multiplex CRISPR/Cas9 leads to reduced lignin deposition and improved forage quality
Source: Front Plant Sci. 2024 Mar 5;15:1363182. doi: 10.3389/fpls.2024.1363182 (PMC10948404; doi:10.3389/fpls.2024.1363182)
Supplement: Supplementary Table 1 — Relative advantage (%) of lignin and nutrition quality in alfalfa Msc3h mutant lines over EV (control). [file Table_1.pdf]

Supplementary file

Mutating alfalfa *COUMARATE 3-HYDROXYLASE* (*MsC3H*) using multiplex RISPR/Cas9 leads to reduced lignin deposition and improved forage quality

Tezera W. Wolabu[1], Kashif Mahmood[1], Fang Chen[4], Ivone Torres-Jerez[1], Michael Udvardi[3], Million Tadege[1], Lili Cong[2], Zengyu Wang[2], Jiangqi Wen[1]

Table S1. Relative advantage (%) of lignin and nutrition quality in alfalfa *Msc3h* mutant lines over EV (control).

Genotype	Relative advantage (%) of each parameter over EV														
	Lignin composition					Forage quality						Minerals			
	S	G	H	Total lignin	S/G ratio	ADF	NDF	TDN	RFV	IVTD MD	CP	P	Ca	K	Mg
Msc3h-013	42.0	33.0	40.0	36.5	14.0	8.0	9.0	9.0	15.0	7.0	30.0	15.0	11.0	7.0	27.5
Msc3h-158	29.0	25.0	20.0	27.0	5.5	7.0	8.0	7.0	14.0	5.0	11.0	7.4	8.0	-6.0	00.0
Msc3h-121	44.0	37.0	25.0	40.0	11.0	11.0	13.0	13.0	23.0	9.0	9.0	18.5	5.5	12.0	23.6
EV	00.0	00.0	00.0	00.0	00.0	00.0	00.0	00.0	00.0	00.0	00.0	00.0	00.0	00.0	00.0

Table S2. **S**equences of primers used in this study

Primer name	Sequence (5' to 3')	Purpose
MsC3H-gRNA1-F	CCATACCCACTTCCCATCAT	For *MsC3H* guide RNA
MsC3H-gRNA1-R	ATGATGGGAAGTGGGTATGG	
MsC3H-gRNA2-F	ACAAAACTCTCAAACATCTA	
MsC3H-gRNA2-R	TAGATGTTTGAGAGTTTTGT	
MsC3H-gRNA3-F	TGAGGTTTTTGATATTGGTG	
MsC3H-gRNA3-R	CACCAATATCAAAAACCTCA	
MsC3H-gRNA4-F	TGGGGTATCATGGAAGAAGC	
MsC3H-gRNA4-R	GCTTCTTCCATGATACCCCA	
L5AD5-F	CGGGTCTCAGGCAGGATGGGCAGTC TGATTGaACAAAGCACCAGTGG	For Golden Gate assembly of the multiplex tRNA-gRNA construction
L3AD5-R	TAGGTCTCCAAACGGATGAGCGAC AGCAAACAAAAAAAAAAGCACCGACTCG	
S5AD5-F	CGGGTCTCAGGCAGGATGGGCAGTCTGATTG	
S3AD5-R	TAGGTCTCCAAACGGATGAGCGACAGCAAAC	
MsC3H-RT-F	CTATTAGTGATTACCTCTTTGTGT	*MsC3H* target region amplification and genotyping
MsC3H-RT-T	TATGAAAAAGTTGTGTGAGATTT	
AtUBQ10-F	CTGCAGGTCGACGAGTCAGTAATA	AtUBQ10 promoter region
AtUBQ10-R	TGTTAATCAGAAAAACTCAGATTAAT	
AtU6-F	GTGATTGTGAGACCGAGAG	AtU6 promoter region
AtU6-R	CTGATAACTCTGATGTGGATAAG	
PPT-F (bar)	GAAGTCCAGCTGCCAGAAAC	PPT selection marker
PPT-R (bar)	AGTCGACCGTGTACGTCTCC	
Hyg-F	AAGGAATCGGTCAATACACTACATGG	Hygromycin selection marker
Hyg-R	AAGACCAATGCGGAGCATATACG	
AtU6-region-RT-F	TTCAAAAGTCCCACATCGC	For vector versions plasmid colony genotyping
sgRNA-scaffold-RT-R	AAGGCGATTAAGTTGGGTAA	
AtU6-region-RT-F2	GTGATTGTGAGACCGAGAG	
sgRNA-scaffold-RT-R2	CTGATAACTCTGATGTGGATAAG	
